# Variations in relative health inequalities: are they a mathematical artefact?

**DOI:** 10.1186/1475-9276-8-32

**Published:** 2009-08-27

**Authors:** Terje A Eikemo, Vera Skalická, Mauricio Avendano

**Affiliations:** 1 Department of Public Health, Erasmus MC, University Medical Centre Rotterdam, P.O. Box 2040, 3000 CA Rotterdam, The Netherlands; 2 SINTEF Health Services Research, Trondheim, Norway, Abelsgt.5, 7465 Trondheim, Norway; 3 Department of Sociology and Political Science, Norwegian University of Science and Technology, Dragvoll University Campus, 7491 Trondheim, Norway; 4 Harvard Center for Population and Development studies, 9 Bow Street, Cambridge, Massachusetts 02138, USA

## Abstract

**Background:**

Substantial research has documented variations in the magnitude of relative socioeconomic differences in health across European countries, and within countries, across different age groups. The aim of this paper is to examine to what extent these variations are determined by differences in the overall rate or prevalence of a health outcome across countries and age-groups in the total population.

**Methods:**

Three surveys (European Social Survey, and two different population census-mortality registry linked longitudinal data) were used. We plotted rates of mortality and prevalence of poor self-rated health against ratios of mortality and morbidity prevalence associated with educational level. We calculated Pearson coefficients to examine the magnitude of correlations.

**Results:**

We found a significant negative correlation between total mortality rates and associated rate ratios of mortality by education in the SEDHA study (r = -0.40, p = 0.04), but not in the HUNT study (r = -0.37, p = 0.06). There was a weaker but significant negative correlation between the prevalence of poor health and associated prevalence ratios by education in the European social survey (r = -0.22, p = 0.00). Correlations increased as underlying prevalence and rates increased, while they were weaker or null at low prevalence or rates.

**Conclusion:**

We found some evidence that the magnitude of relative inequalities in mortality and morbidity is negatively correlated with underlying morbidity prevalence and mortality rates. Although correlations are moderate, underlying morbidity prevalence and mortality rates should be taken into account in the interpretation of variations in relative health inequalities among populations.

## Introduction

It has been pointed out that variations in health inequalities can be explained by a mathematical rule rather than by substantial interpretations. [1] This heuristic mathematical rule (HRX) suggests that all measures of differences between rates of experiencing binary outcomes, as well as all measures that are functions of binary outcomes, appear to change in one manner or another as there occurs a change in the overall prevalence of an outcome. In previous studies, it has been shown that rate ratios of health vary by country, but also by age, gender, or many other demographic characteristics. It is known, that as death rates increase, the relative risk becomes smaller, and as death rates decrease, they become larger. For example, rate ratios of inequalities for two diseases that have the same level of inequality but one of them is twice as common that the other one will yield different rate ratios. Therefore, in such cases, we need to carefully interpret variations in estimates of relative health inequalities. If variations in health inequalities are mainly driven by a mathematical artefact, not only would conclusions from previous studies providing substantial explanations be questionable, but we would also have to look for new strategies to assess and interpret variations in relative health inequalities across countries or population sub-groups.

The aim of this study is to examine to what extent education-associated ratios for mortality and prevalence of a health outcome are correlated with underlying morbidity prevalence and mortality rates. We expect our study to be a first attempt to investigate these issues by examining how strong the correlation is between absolute levels of the health outcome and relative measures of health inequalities. Our first hypothesis is the following:*"The magnitude of prevalence ratios between high and low educated men and women from 6 age cohorts in 23 European nations is to a great extent dependent on the total prevalence of morbidity for these men and women"*. According to the HRX, this implies that a linear relationship will occur between the prevalence ratios and morbidity prevalence, in which the lowest point of the graph will correspond to lower morbidity prevalence. Our second hypothesis deals with the relationship between rate ratios and total mortality rates, in which age cohorts are the unit of the analysis: *"The magnitude of rate ratios between age cohorts is to a great extent dependent on the total prevalence of mortality within each age group"*. According to the HRX, this would mean that the rate ratio would be highest where the total mortality rate is low, and would subsequently decrease gradually with higher mortality rates.

## Methods

The first hypothesis was tested using cross-sectional data from the first, second and third wave of the European Social Survey (ESS), fielded in 2002, 2004, and 2006 which comprised 108 835 individuals from 23 European countries after list wise deletion. Poor health was measured as 'less than good health' from an original five point scaled variable providing information on the respondents' general physical and mental health. The measure of education was based on a variable describing full-time education in years. For each country, sex and 6 age-cohorts (people born in the 30 s, 40 s, 50 s, 60 s, 70 s and 80 s), we standardised the continuous scale of educational years, such that the average was equal to 0 and the standard deviation equal to 1 year of education. We also reverted this variable by multiplying it with a factor of -1, such that higher values corresponded with lower educational levels. Next, the standardised variable was introduced as an independent variable in a Poisson model, controlled for age and ESS-round, with poor health as the dependent variable. Because it has been demonstrated that trends in odds ratios may overstate or understate trends in relative inequality in health when the outcome is of relatively high prevalence [2], we have chosen the prevalence ratio as our relative measure for health inequality. Based on the recommendations of Spiegelman & Hertzmark [3] the prevalence ratios (PR) were computed using SAS PROC GENMOD's Poisson regression capability with the robust variance, since it is well known that the log-binomial model is less numerically stable than the logistic model. The PR should be interpreted as the health difference between people with average years of education and those whose number of year of education is one standard deviation below the national average. We thereby take into account the extent of variations of reported years of education in different countries. On this basis PRs were calculated, which we in turn compared to the corresponding total prevalence rates.

The second research question was examined using data from the SEDHA and HUNT study. The HUNT study (1984–1986), a cross-sectional health survey based on a total county population in Norwegian Nord-Trondelag county (88.1% response rate), was linked to 1985 census data on education, and to the national death registry in 2003. The utilised mortality follow-up sample comprises data on 61 059 men and women aged 20 years or older (1 003 387 person-years). Education was measured on eight levels of educational attainment. For each 5 years age group, low education was defined on 60–75% cut off point of the educational distribution. Hazard rates of low versus high education calculated for each age group were compared to total mortality rates.

The SEDHA study comprises longitudinal data on mortality by educational level, sex, and 5-year age group for 10 European populations.[4] Participants were enumerated during a census in the early 1990s and followed up for different periods. Most studies covered the entire national population, except Madrid (regional), Barcelona and Turin (urban), Switzerland (population living in German-speaking areas), and England/Wales (1% representative sample of the population). Studies included individuals aged 30 years (age specified at the start of follow-up), except in Denmark, where data on education were not available for those aged 70 years. Educational level was first coded according to national classification schemes and was subsequently reclassified into 3 equivalent categories so that the proportion of individuals with a low educational level was similar across populations within each age-group. Although small variations remained, the proportion of low educated men and women was 60–70% in most age-groups. 5-year age group specific rate ratios that compared the lowest educated with the rest of the population within the same age group were calculated using Poisson regression.

## Results

**Figure 1:**
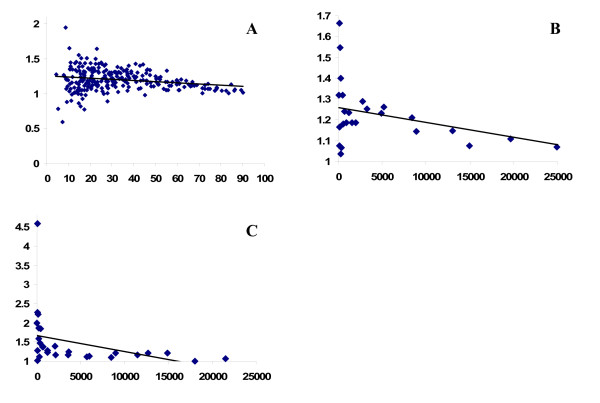
**Correlations between morbidity prevalence and prevalence ratio of reporting poor health (A) and between mortality rate and relative risk (B and C)**. A: Morbidity prevalence of poor health versus prevalence ratios of 1 standard deviation decrease of full-time educational years for men and women in 6 age cohorts belonging to 23 European countries. Data source is the European Social Survey from 2002, 2004 and 2006 (merged). B: Rate ratio of mortality according to educational level plotted against the respective mortality rates by 5-year age group, aged 30 years or older in the SEDHA study. C: Rate ratio of mortality according to educational level plotted against the respective mortality rates by 5-year age group in the HUNT study.

Figure 1A shows the relationship between age-adjusted morbidity prevalence ratios and prevalence rates for men and women within 6 age cohorts in 23 European countries. There was a significant negative correlation between the prevalence of poor health and associated prevalence ratios by education (r = -0.22, p = 0.00). The figure shows that this association became stronger as the prevalence of poor-health increased. For example, the correlation coefficient calculated only among populations with prevalence of 50% or higher was -0.67 (p = 0.00), while there was no correlation among populations with prevalence below 50% (r = 0.08, p = 0.26). In sensitivity analysis, we found roughly the same pattern of correlations when applying a categorical dichotomous (cut-off at lower secondary education or less) version of the education variable (r = -0.28, p = 0.00).

Figure 1B plots rate ratios of mortality according to educational level against total mortality rates by 5-year age group, separately for men and women aged 30 years or older. In support of the heuristic rule X, there was a significant negative correlation between total mortality and the size of the rate ratio (r = -0.41, p = 0.04). Relative risks of mortality by educational level were highest at low levels of overall mortality; they decreased gradually and were lowest among those with highest total mortality. The association increased as rates of mortality increased. For example, among populations above median mortality, the correlation was -0.78 (p = 0.00), while the correlation among populations below the median was small and non-significant (r = -0.19, p = 0.51).

Figure 1C shows mortality rate ratios of low versus high education plotted against the respective mortality rates for each 5-year age group separately for men and women in the HUNT study. The correlation between total mortality rates and rate ratios of mortality by education was not significant (r = -0.37, p = 0.06). However, the correlation above median mortality was negative and significant (r = -0.58, p = 0.03), while the correlation below the median was non-significant (r = -0.34, p = 0.24).

Overall, results provide some support for the hypothesis that total mortality levels are associated with the magnitude of relative risks.

## Discussion

We found that the magnitude of health inequalities is associated with underlying health levels. This association is stronger at higher levels of mortality or morbidity, while it is less marked at lower morbidity prevalence and mortality rates. Houweling [5] showed that both absolute and relative inequality measures can be meaningful for monitoring inequalities, provided that the overall level of the outcome is taken into account. Our results provide further support for this view, particularly for populations and age groups that experience relatively high morbidity and mortality.

Although the correlation between inequalities in mortality and total mortality rates was of moderate size, our findings do raise questions about the interpretation of variations in mortality inequalities across countries or age-groups with different levels of underlying mortality. At least two interpretations of our findings should be considered. On the one hand, our results might be interpreted as to indicate that countries or age-groups with lower mortality tend to experience larger inequalities in mortality. Although this is plausible, it is unlikely that countries that have been successful in reducing mortality rates would be less successful in decreasing the magnitude of inequalities in mortality. A second alternative explanation calls upon the mathematical limits inherent to measures of relative effect. [5] Our findings suggest that these limits might yield relative risks as partly misleading if used as single measures to compare the magnitude of mortality inequalities across populations or age-groups with different underlying mortality levels. The main implication of this finding is that measures of relative effect should preferably be interpreted hand-by-hand with total rates and estimates of absolutes inequalities in mortality (e.g., rate differences). [5]

We found a significant negative correlation between the prevalence of poor self-rated health and the magnitude of education-related inequalities in this outcome. This correlation was weaker than for mortality, suggesting that the impact of absolute morbidity prevalence levels on the magnitude of prevalence inequalities might be less dramatic than for mortality. On the other hand, we found that correlations were strong among populations with high prevalence of poor self-rated health. Thus, our results suggest that estimates of relative health inequalities should also be interpreted together with estimates of absolute effect that are sensitive to underlying morbidity prevalence levels in the population.

In conclusion, our findings support the view that absolute morbidity prevalence and mortality levels should be considered in the interpretation of variations in relative inequalities in health across countries or age-groups. Although correlations are of moderate size and may not fully explain all variation, findings from previous studies that compare health inequalities across countries and age-groups should be re-interpreted in the light of our findings.

## Competing interests

The authors declare that they have no competing interests.

## Authors' contributions

TAE coordinated the paper, conducted the data analysis and interpretation of the European Social Survey, and contributed to the write up of the paper. VS conducted the data analysis and interpretation of the HUNT study, and contributed to the write up of the paper. MA conducted the data analysis and interpretation of the SEDHA study, and contributed to the write up of the paper. All authors read and approved the final manuscript.
